# Cervical spinal cord compression in children and adolescents with 22q11.2 deletion syndrome

**DOI:** 10.1016/j.bas.2026.107480

**Published:** 2026-10-14

**Authors:** Jette A. Boxem, Michiel L. Houben, Malcolm L. Ecker, Erin S. Schwartz, Tom P. Schlösser, E. Zackai, T. Blaine Crowley, Aebele B. Mink van der Molen, Donna M. McDonald-McGinn, Alexander M. Tucker

**Affiliations:** a Dept. of Plastic Surgery, University Medical Center Utrecht, the Netherlands; b Dept. of Genetics, Children's Hospital of Philadelphia, United States of America; c Dept of Pediatrics, University Medical Center Utrecht, the Netherlands; d Div. of Orthopedic Surgery, Children's Hospital of Philadelphia, United States of America; e Div. of Neurosurgery, Children's Hospital of Philadelphia, United States of America; f Div. of Neuroradiology, Children's Hospital of Philadelphia, United States of America; g Dept. of Orthopaedic Surgery, University Medical Center Utrecht, the Netherlands

**Keywords:** Cervical spine, Cord compression, Myelopathy, 22q11 deletion syndrome, 22q11DS

## Abstract

**Introduction:**

Cervical spine anomalies are common in patients with 22q11.2 deletion syndrome (22q11DS), but the clinical course and risk of spinal cord compression remain poorly defined in this population. Current guidelines recommend radiographic cervical spine screening for cervical instability around 4 years of age, yet cases of compression are often detected later. This study aims to characterize the clinical and radiological presentation of patients with 22q11DS who develop cord compression to aid early recognition and to improve awareness.

**Research question:**

What are the clinical trajectories and radiological features of 22q11DS patients who develop cervical spinal cord compression?

**Methods:**

This is a retrospective multicenter case-series of patients with 22q11DS and cervical spinal cord compression requiring surgery. Clinical and radiological findings were reviewed of routine screening and pre-operative assessment. All 22q11 patients older than four years underwent routine cervical spine screening, in accordance with current clinical guidelines for 22q11DS.

**Results:**

5 patients were identified with cervical spinal cord compression**.** Three presented with neurological symptoms, two of which could be attributed to cord compression, while two patients were asymptomatic. Four of five patients demonstrated no evidence of cervical instability on cervical lateral radiographs at their initial evaluation (age ranged 2-13 years).

**Discussion and conclusion:**

Most patients with 22q11DS and cervical myelopathy presented in adolescence and did not show signs of instability during initial evaluation for cervical instability. These findings suggest that continued vigilance beyond childhood is warranted, with particular attention to signs of cervical myelopathy during adolescence to facilitate timely diagnosis and intervention.

## Highlights

• Cervical cord compression is a rare but relevant complication in 22q11DS.
• Early cervical imaging may not predict later cord compression.
• Neurological follow-up beyond childhood is warranted in 22q11DS.
• First report of multiple 22q11DS patients with cervical cord compression.

## 1 Introduction

22q11.2 deletion syndrome (22q11DS) is a multisystem condition with a broad phenotypic spectrum, including physical, cognitive and behavioral symptoms, that occur in 1 in 2148 live births (McDonald-McGinn et al., 2015; Blagojevic et al., 2021). Congenital cervical spine anomalies are frequently observed in this population. Previous studies demonstrated that 90.5-100% of the patients have at least one congenital anomaly of the cervical spine (Homans et al., 2018; Ricchetti et al., 2008). These anomalies are not unique to 22q11DS, but are also observed in other syndromes, such as trisomy 21, achondroplasia and Klippel-Feil syndrome (Courvoisier, 2023; Warner et al., 2021).

Despite the high prevalence of congenital cervical spine anomalies, the clinical significance of many of the anomalies remains unclear. Upper cervical instability or basilar impression may raise concerns for bulbar symptoms, myelopathy or progressive neurological deficits (Ricchetti et al., 2008; Courvoisier, 2023; Hensinger, 1991). Only four cases in the literature have described cervical spinal cord compression in patients with 22q11DS (Ricchetti et al., 2008; Konen et al., 2008; Boot et al., 2015; Bolland et al., 2000). Therefore, the characteristics of patients with 22q11DS at risk for spinal cord compression remain poorly defined.

In the reported cases, affected patients had compression of the cord at the level of the upper cervical spine (Ricchetti et al., 2008; Konen et al., 2008; Boot et al., 2015; Bolland et al., 2000). Symptoms included gait disturbance, motor weakness and hyperreflexia. One case described upper extremity weakness and shoulder hypotonia, but this was not linked to the cord compression (Bolland et al., 2000).

Current 22q11DS guidelines recommend a one-time screening for cervical spine instability by dynamic cervical radiographic imaging in flexion and extension around 4 years of age (Óskarsdóttir et al., 2023). Severe instability or clinical signs of myelopathy require urgent referral for neurological work-up to prevent permanent damage (Bassett et al., 2011).

In other syndromes with comparable congenital cervical spine anomalies, management strategies emphasize that treatment decisions are mostly guided by neurological signs and symptoms rather than by anatomical abnormalities alone. For example, in children with Down syndrome, the American Academy of Pediatrics (AAP) does not recommend repeated radiological surveillance for atlantoaxial instability in patients without symptoms, underscoring the limited clinical relevance of imaging in asymptomatic patients (Bull et al., 2022). As early recognition is essential to prevent permanent damage, identifying those patients with 22q11DS who are at highest risk to develop spinal cord compression is essential to guide clinical recognition and management.

We hypothesize that cord compression in the 22q11DS population may develop over time and may be absent on routine radiographic imaging in early childhood. By describing the clinical course and radiological presentation of affected patients that underwent surgery, this case series aims to support earlier recognition and awareness of this potentially severe condition in patients with 22q11DS.

## 2 Methods

Patients included in this study were enrolled in an IRB approved protocol and provided informed consent. This case series was conducted with consideration of established methodological recommendations for the reporting of uncontrolled case series (Kempen, 2011).

### 2.1 Patient population

This multicenter case series included patients followed within the 22q11 specialty centers at Wilhelmina Children's Hospital, Utrecht, The Netherlands, and the Children's Hospital of Philadelphia, USA. Patients with a history of cervical cord compression were identified in retrospect from the original 22q11DS databases in both hospitals by neurosurgical intervention code between 1992 – June 2025 or decompressive surgery described in the patient file (Fig. 1) Five patients underwent cervical decompressive surgery. All had cervical spinal cord compression confirmed by magnetic resonance imaging (MRI) and laboratory confirmed diagnosis of a standard LCR22A-LCR22D chromosome 22q11.2 deletion.

**Fig. 1:**
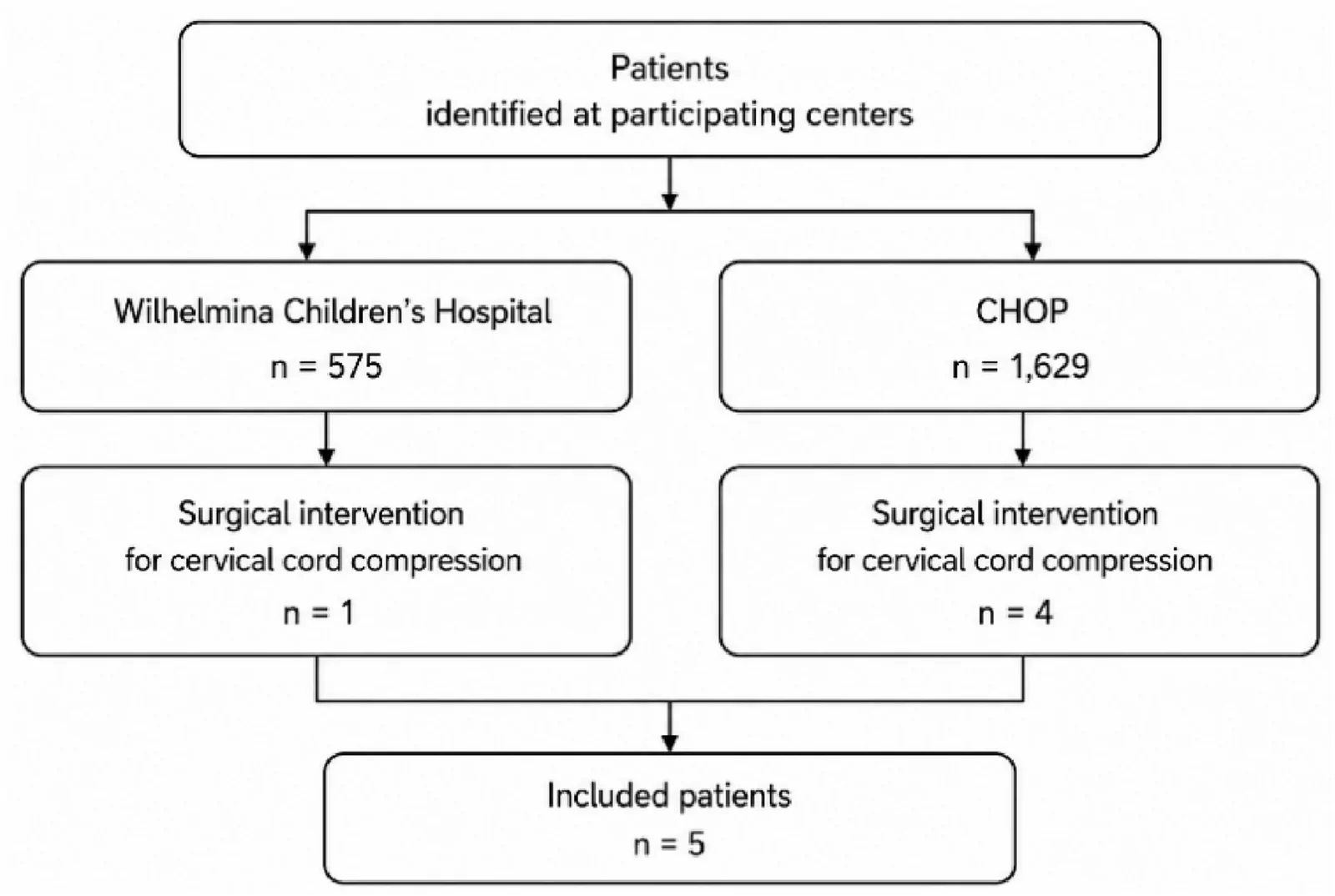
Flowchart of patient inclusion.

### 2.2 Radiological assessment

Routine cervical screening for instability and preoperative assessment including conventional radiographs of the cervical spine, computed tomography (CT) and MRI were reviewed for occipitoatlantal and/or atlantoaxial instability, congenital cervical anomalies and cord compression. Radiological imaging of all patients was independently reviewed by a radiologist, neurosurgeon and orthopedic surgeon. Atlantodental interval (ADI) was measured between the anterior surface of the dens and the posterior surface of the C1 anterior arch. In this population, an interval >4 mm in children on flexion-extension radiographs suggested instability and warranted further evaluation (Hensinger, 1991; Copley and Dormans, 1998). Occipitoatlantal instability was assessed by the translation of the anterior surface of the occipital condyles to the posterior surface of the anterior arch C1 (Ricchetti et al., 2008).

The clivus-canal-angle (CCA), was measured as the angle between the clivus line and posterior axial line drawn along the lower third of the clivus, from the basicranial synchondrosis to the basion. For presence of basilar invagination, a line was drawn from the basicranial synchondrosis to the odontoid process (Henderson et al., 2018). Normal CCA is 135-175°, with angles ≤135 suggesting atlantoaxial impaction and possibly correlated with myelopathy (Morishita et al., 2009).

## 3 Case reports

### 3.1 Patient 1

Patient 1 is a male with a medical history notable for 22q11DS diagnosed at age 2 years, developmental delay, cognitive deficits and microcephaly. Additional genetic testing revealed a variant in the *TANGO2* gene on the intact chromosome 22q11.2 allele consistent with *TANGO2* Deficiency Disorder (TDD), but not associated with cervical spine anomalies.

The patient presented with seizure-like activity at age 2 years. His neurologic exam revealed hypertonia, upper extremities greater than the lower extremities, hyperreflexia, positive Babinsky signs bilaterally, positive Hoffman's test and a wide-based gait. EEG monitoring did not demonstrate any epileptiform abnormalities and X-ray showed no instability. CT showed incomplete fusion of the anterior and posterior arch of C1, with a decreased anteroposterior dimension, C2-C3 failure of segmentation and a normal CCA. Slight indentation of the posterior cervical spinal cord by the posterior arch of C1, without signal abnormality was seen on MRI (Fig. 2). He underwent planned C1 laminectomy (age 2 years), with immediate improvement of motor signals in the upper extremities. His postoperative neurological exam was normal and MRI showed adequate space for the cord without signal abnormalities However, he continued to have occasional spells of unknown etiology.

**Fig. 2:**
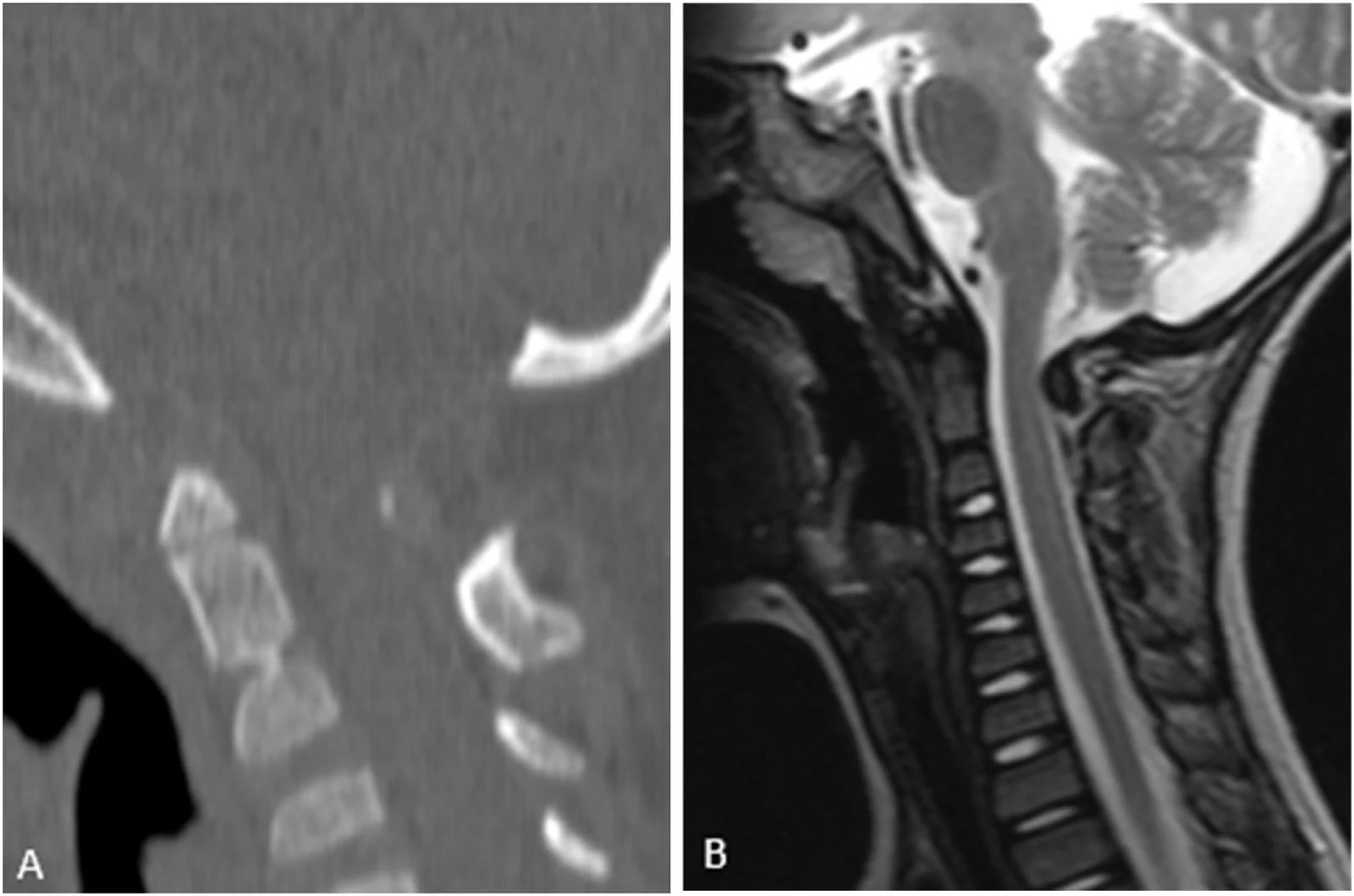
Patient 1 (age at imaging 2yr). (A) CT-imaging: failure of segmentation C2-3 and incomplete ossification of the C1 anterior and posterior arch. (B) T2 MRI-imaging: posterior indentation of the cord at the level of C1.

### 3.2 Patient 2

Patient 2 is a male with a medical history notable for 22q11DS diagnosed at age 2 years, congenital heart defect (CHD), right renal agenesis, velopharyngeal insufficiency, scoliosis and camptodactyly.

His cervical spine was evaluated radiographically at the age of 4 years. There was no evidence of occipitoatlantal or atlantoaxial instability. However, platybasia, incomplete fusion anterior and posterior arch C1, hypoplastic occipital condyles and C2-C3 failure of segmentation were documented. Physical examination was unremarkable. At age 8 years, he presented with abnormal gait, a head tilt, and neck stiffness but neurologic exam did not reveal any focal deficits and MRI did not reveal any additional abnormalities.

At age 13 years, a full spine MRI obtained for screening for neural axis abnormalities associated with scoliosis revealed narrowing at the craniocervical junction and indentation of the cord due to upward displacement and dorsiflexion of the odontoid and posterior tenting of the dura (Fig. 3). His neurological exam, including strength, tone, deep tendon reflexes (DTR) and gait was normal. Flexion/extension radiographs showed mild occipitoatlantal instability and normal ADI. CT showed basilar invagination and an acute CCA (126°). To prevent further injury, he underwent planned C0-C3 instrumented fusion and C1 laminectomy at age 14 years. Postoperatively he developed hypocalcemia which normalized following calcium supplementation, his neurological exam remained normal and CT showed good alignment.

**Fig. 3:**
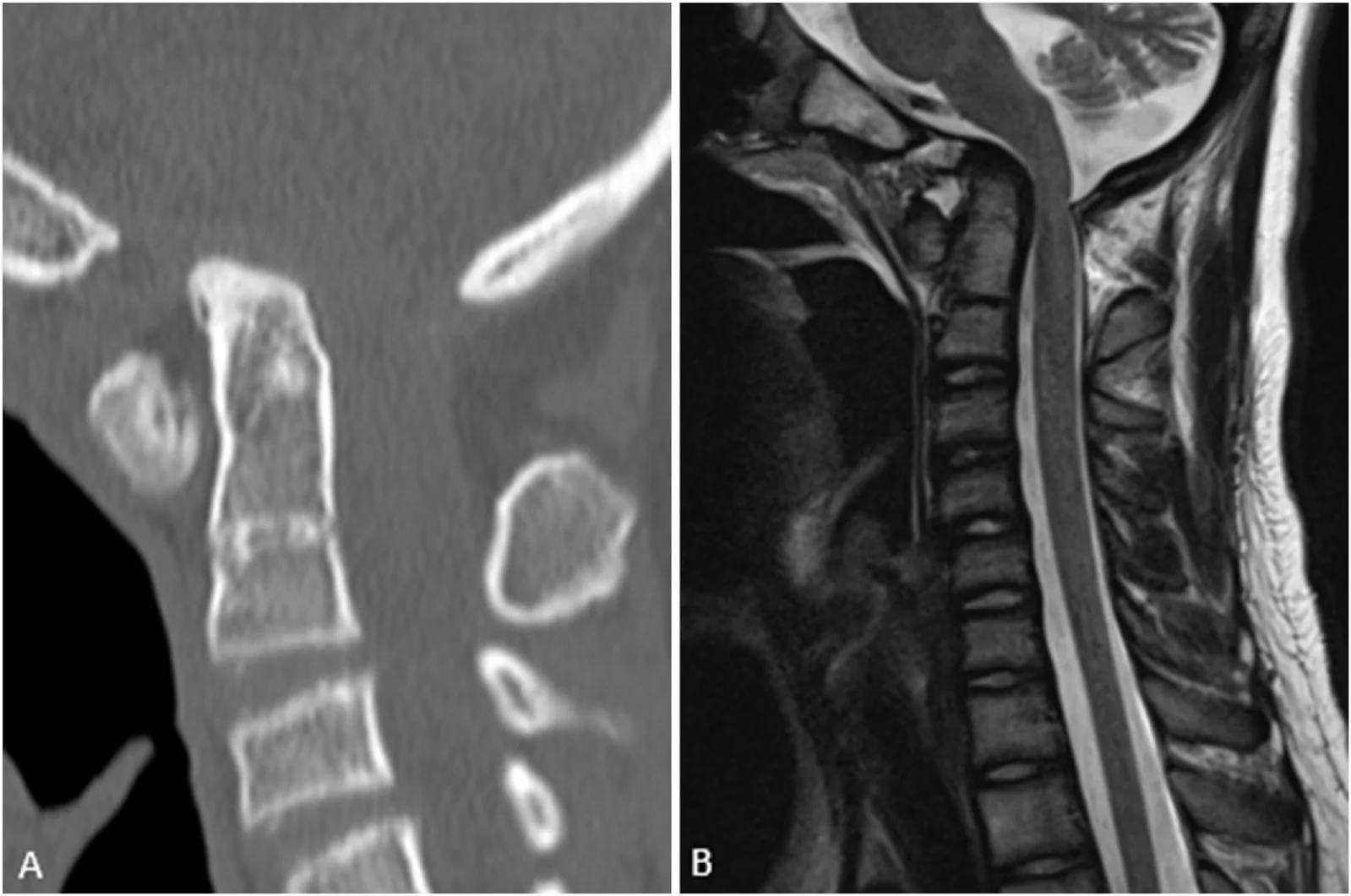
Patient 2 (age 13yr). (A) CT-imaging: C2-3 failure of segmentation, open posterior arch C1, basilar invagination. (B) T2 MRI-imaging: cord compression at the craniocervical junction.

### 3.3 Patient 3

Patient 3 is a male with a medical history notable for 22q11DS diagnosed at age 4 years, CHD, scoliosis and constipation.

At age 6 years, a CT was obtained after detection of possible occipitoatlantal instability on flexion/extension radiographs with a 5 mm translation on extension, which was absent at subsequent radiographs. ADI remained normal. The CT showed incomplete fusion anterior and posterior arch C1, failure of segmentation C2-3, bifid posterior spinous processes C2 and C3. Furthermore, an MRI of the spine was obtained to rule out tethered cord due to his longstanding constipation and encopresis, which was negative for tethered cord but demonstrated cervical stenosis without cord compression. His cervical spine was routinely monitored radiographically at age 8 and 11 years, without evidence of subluxation.

At age 13 years, he presented with severe leg cramps and the inability to walk long distances due to leg pain. Physical examination showed hyperreflexia in the lower extremities and a wide-based gait. Repeated imaging revealed increased cervical stenosis and posterior compression of the cord with cord edema by the anteriorly displaced posterior arch of C1 and an acute CCA (134°) (Fig. 4). He underwent C1-C3 laminectomy at age 13 years to decompress the cord. Afterward, he developed worsening of the cervical instability due to anterior subluxation of C3 on C4 detected on CT after presenting with headaches and neck pain requiring a posterior fusion C0-C5 3 months after the initial laminectomy. He underwent multiple revisions at 14 years of age due to construct failure including anterior fusion and extension of the posterior instrumentation. At age 15 years, his neurological exam was normal and postoperative CT showed adequate cervical fusion and hardware positioning.

**Fig. 4:**
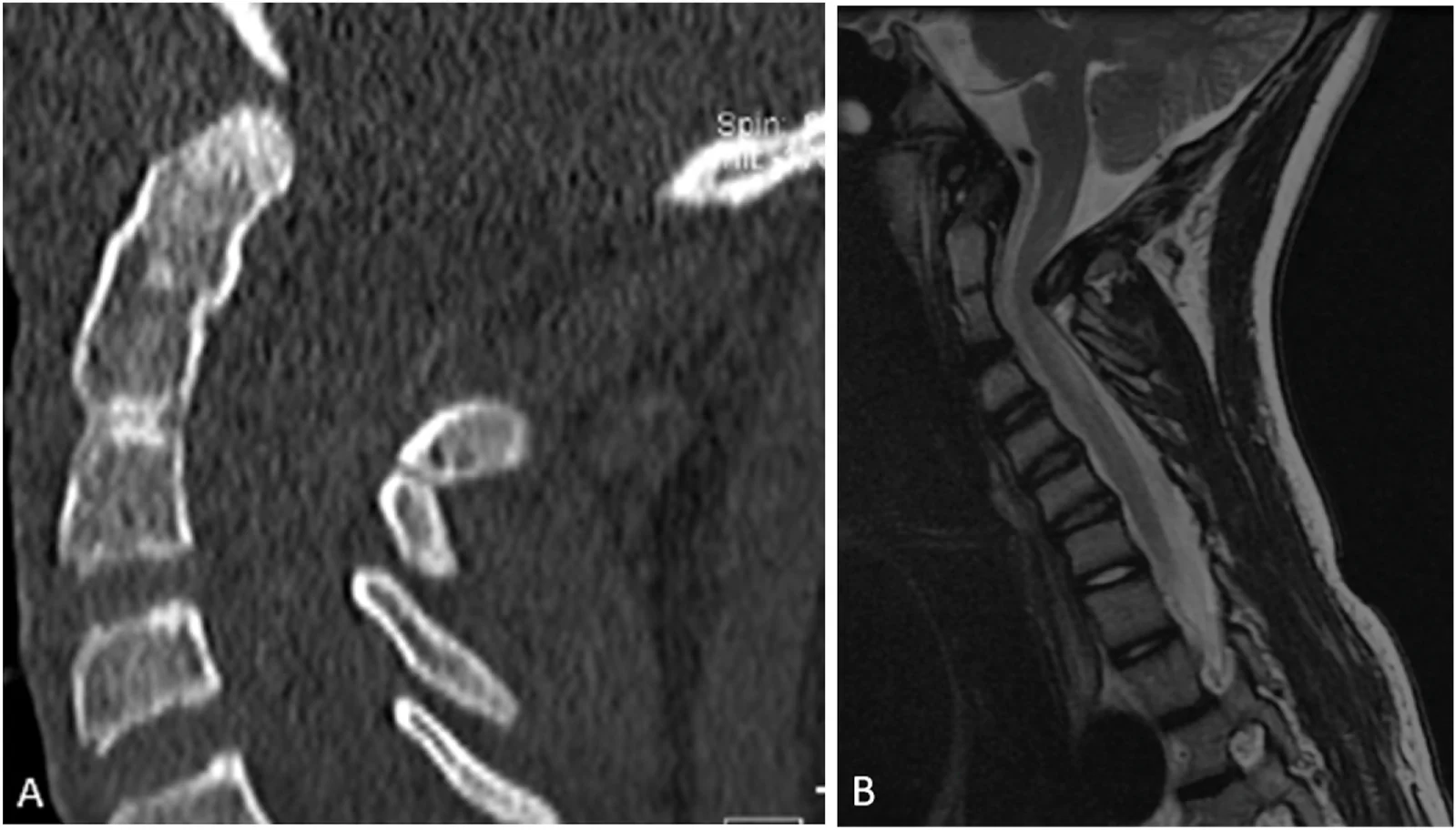
Patient 3 (age 13yr). (A) CT-imaging: incomplete fusion anterior and posterior arch C1, C2-3 failure of segmentation. (B) T2 MRI-imaging: cord compression at the level of C2.

### 3.4 Patient 4

Patient 4 is a male with a medical history notable for 22q11DS diagnosed at age 13 years, camptodactyly and syndactyly, leg length discrepancy, Juvenile Idiopathic Arthritis (JIA), osteoporosis due to JIA medication and avascular necrosis of both femoral heads.

He was referred to a 22q specialty center at age 13 years for routine evaluation. Imaging revealed platybasia, incomplete fusion anterior and posterior arch C1, C0-C1 failure of segmentation and basilary impression, without signs of upper cervical instability. Additional findings include thoracolumbar scoliosis. Aside from his curvature, physical examination, including neurologic exam was normal.

At the age of 15 years, he experienced progressive gait disturbances, increased tripping and weakness predominantly affecting the right leg. Physical examination revealed significant weakness in the lower extremities and hyperreflexia. MRI demonstrated an acute CCA, pronounced basilar invagination with cord compression (Fig. 5). The patient underwent emergency transoral odontoid/C2 body resection and posterior fusion C0-C4. Postoperative radiographs revealed adequate alignment and he recovered without complications.

**Fig. 5:**
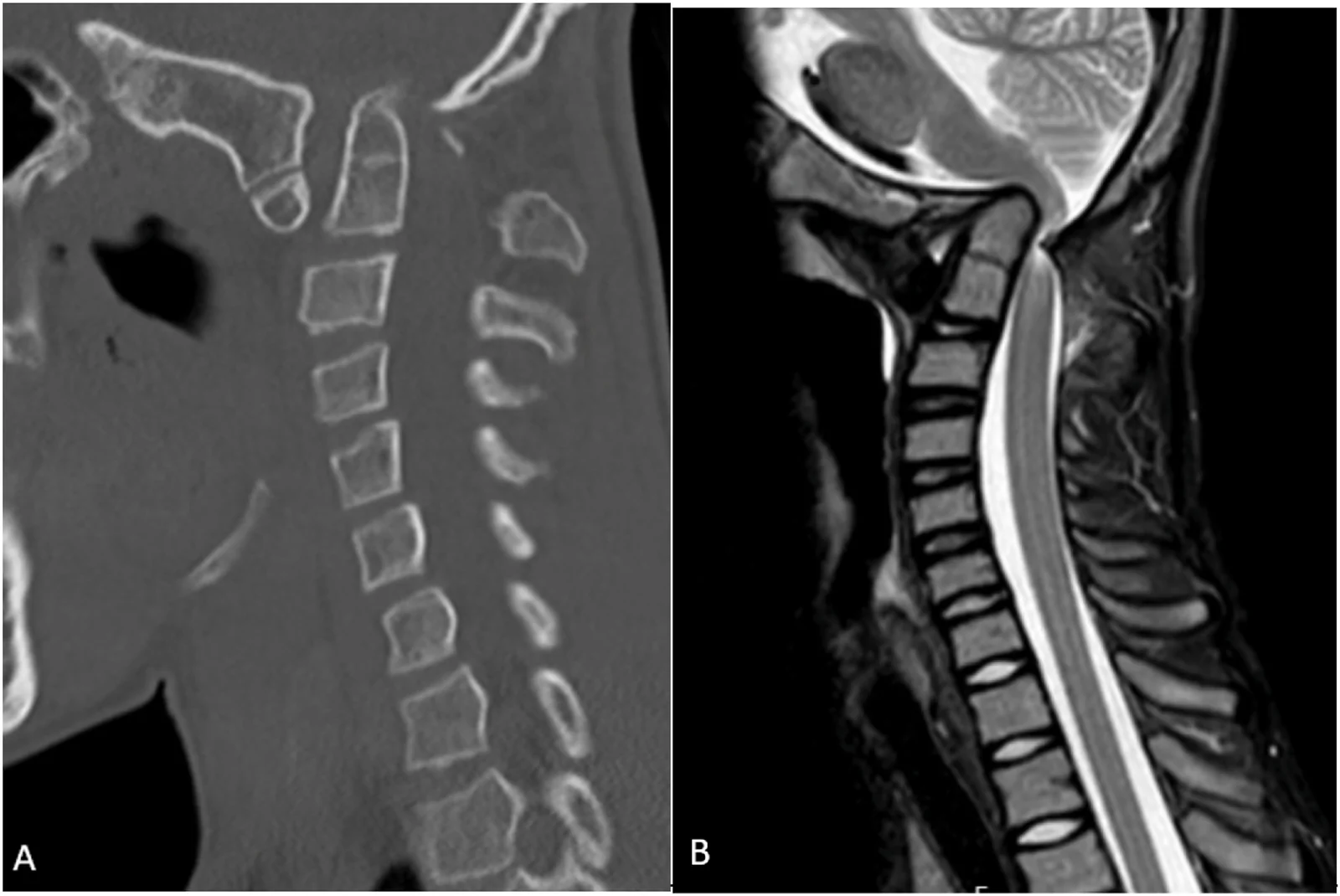
Patient 4 (age 15yr). (A) CT-imaging: basilar invagination, incomplete posterior arch C1. (B) T2 MRI-imaging: severe cord compression at craniocervical junction.

### 3.5 Patient 5

Patient 5 is a female with a medical history notable for 22q11DS diagnosed at age 4 years, CHD, short stature and developmental delay.

By protocol, she underwent screening at age 4 years, without upper cervical instability. Her physical examination was unremarkable. She underwent cardiac surgery, after which she had a seizure.

At 6 years of age an MRI of the brain was obtained for evaluation of her previous seizure which demonstrated significant narrowing at the occipitocervical junction with ventral and dorsal compression at the level of the C2 synchondrosis as well as myelomalacia. CT-imaging showed anterior positioning of the posterior arch of C1, with also incomplete fusion of the posterior and anterior arch of C1 (Fig. 6). At that time, her neurological exam, including strength, tone and DTR's was still unremarkable. She underwent C1 laminectomy at age 7 years to prevent further injury. She recovered without complications and the postoperative MRI showed adequate cord space.

**Fig. 6:**
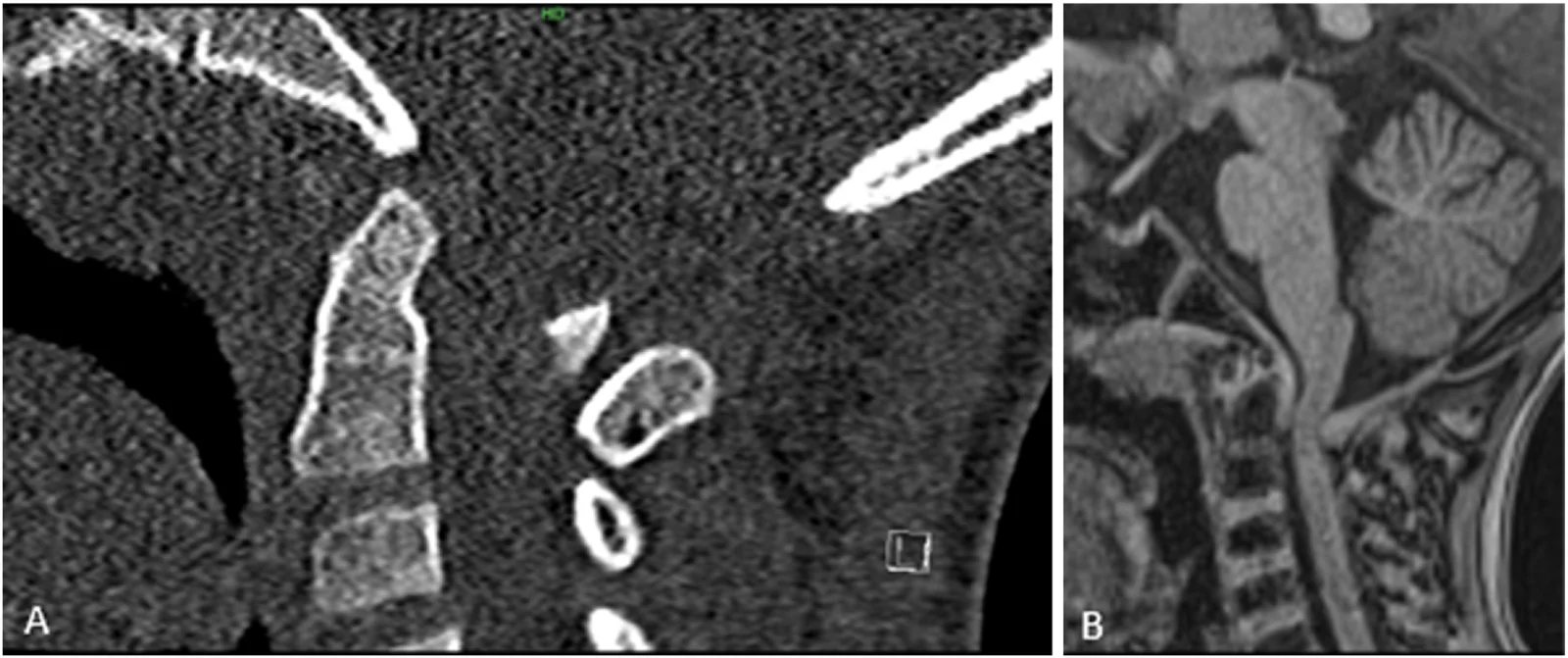
Patient 5 (age 6yr). (A) CT-imaging: incomplete fusion anterior arch C1, anterior positioning posterior arch C1. (B) T1 MRI-imaging: cord compression at craniocervical junction.

## 4 Discussion

This study is the first to describe a series of 22q11DS patients (n = 5) with upper cervical spinal cord compression undergoing surgical decompression. Three patients presented with neurological symptoms, two of which could be attributed to spinal cord compression, while two patients were asymptomatic. All patients older than four years (n = 4) underwent previous routine cervical spine screening, in accordance with current guidelines for 22q11DS (Óskarsdóttir et al., 2023; Bassett et al., 2011) at the ages of 4, 4, 6 and 13 years (Table 1). Three of four patients showed no signs of cervical instability on radiographs at previous evaluation. These findings suggest that instability may develop over time, indicating that screening in early childhood may be insufficient to detect these manifestations that may ultimately require surgical intervention at later age.

**Table 1:** Series of 22q11DS patients with cervical spinal cord compression and ages at specific clinical stages (in years).

Pt	Sex	Age at 22q11DS diagnosis	1st ORT consultation age	Follow-up ages preoperatively	Age at RAD diagnosis	Age at spine surgery	Age at latest follow-up
1	M	2	2	-	2	2	17
2	M	2	4	8, 11, 13	13	14	15
3	M	4	6	8, 11, 13	13	13	15
4	M	13	13	15	15	15	22
5	F	4	4	6	6	7	12

All congenital anomalies observed in the presented patients are known to be associated with 22q11DS (Homans et al., 2018). Although these abnormalities occur at relatively high frequencies, they are not unique to 22q11DS. Clinical management of cervical spine anomalies differ between syndromic or acquired conditions, largely reflecting variation in the associated risk of developing instability. In contrast to conditions like Down syndrome, where ligamentous laxity and atlantoaxial instability are frequently recognized in early childhood via established screening protocols, our findings highlight a more unpredictable chronological pattern in 22q11DS. While roughly 15-30% of patients with Down syndrome demonstrate radiographic instability early on—often remaining stable or improving with skeletal maturity—our cohort demonstrates that normal early childhood screening in 22q11DS does not preclude the late development of severe, symptomatic spinal cord compression. The fact that three of our four screened patients initially had normal radiographic alignment underlines that, unlike the relatively static or early-manifesting instability of other syndromes, cervical risk in 22q11DS can be progressive and deceptive. This fundamentally challenges the efficacy of a single, time-limited screening window in early childhood, as included in the current guideline.

This study has several limitations. First, this is a retrospective, multicenter case series of patients who underwent surgical decompression, rather than a cohort of all patients with cervical spinal cord compression or stenosis. Consequently, non-surgical cases were not described. Second, incomplete follow-up and routine cervical imaging into adulthood preclude an accurate epidemiological evaluation. Third, the inclusion of five patients limits generalizability and may not capture all features associated with cervical cord compression in this population. Not all patients with the shared findings described will develop spinal cord compression, while some without them may still be at risk. Nonetheless, this pathology appears rare but clinically relevant, with potentially severe consequences.

## 5 Conclusion

Spinal cord compression in patients with 22q11DS appears to be a rare but clinically relevant complication that may develop over time, without signs of upper cervical spine instability in early childhood. Although early imaging detects congenital abnormalities, it may not predict later instability or symptomatic cord compression. These findings suggest that increased awareness and continued vigilance beyond childhood is warranted, with particular attention to neurological examination during adolescence. Early recognition of myelopathy by any specialist involved in the care for 22q11.2DS patients may help reduce the risk of permanent neurological deficits. Finally, awareness of these rare associations would benefit from inclusion in both the pediatric and adult 22q11DS healthcare recommendations, irrespective of structural radiographic cervical screening.

## Ethics declaration

Written informed consent to take part in the study and to publish the article has been obtained from all participants or their legal representatives. The privacy rights of participants have been observed.

This study was performed in compliance with relevant laws, regulatory frameworks and guidelines where the research took place. This study was approved by the CHOP IRB A. (Approval No. 07-005352)

This research follows the CARE guidelines and the CARE checklist.

## Declaration of competing interest

The authors declare that they have no known competing financial interests or personal relationships that could have appeared to influence the work reported in this paper.

## References

[bib1] Bassett A.S., McDonald-McGinn D.M., Devriendt K., Digilio M.C., Goldenberg P., Habel A. (2011). Practical guidelines for managing patients with 22q11.2 deletion syndrome. J. Pediatr..

[bib2] Blagojevic C., Heung T., Theriault M., Tomita-Mitchell A., Chakraborty P., Kernohan K. (2021). Estimate of the contemporary live-birth prevalence of recurrent 22q11.2 deletions: a cross-sectional analysis from population-based newborn screening. CMAJ Open.

[bib3] Bolland E., Manzur A.Y., Milward T.M., Muntoni F. (2000). Velocardiofacial syndrome associated with atrophy of the shoulder girdle muscles and cervicomedullary narrowing. Eur. J. Paediatr. Neurol..

[bib4] Boot E., Butcher N.J., van Amelsvoort T.A., Lang A.E., Marras C., Pondal M. (2015). Movement disorders and other motor abnormalities in adults with 22q11.2 deletion syndrome. Am. J. Med. Genet..

[bib5] Bull M.J., Trotter T., Santoro S.L., Christensen C., Grout R.W. (2022). Health supervision for children and adolescents with Down syndrome. Pediatrics.

[bib6] Copley L.A., Dormans J.P. (1998). Cervical spine disorders in infants and children. J. Am. Acad. Orthop. Surg..

[bib7] Courvoisier A. (2023). Congenital cervical spinal deformities. Orthop. Traumatol.: Sur. Res..

[bib8] Henderson F.C., Henderson F.C., Wilson W.A., Mark A.S., Koby M. (2018). Utility of the clivo-axial angle in assessing brainstem deformity: pilot study and literature review. Neurosurg. Rev..

[bib9] Hensinger R.N. (1991). Congenital anomalies of the cervical spine. Clin. Orthop. Relat. Res..

[bib10] Homans J.F., Tromp I.N., Colo D., Schlösser T.P.C., Kruyt M.C., Deeney V.F.X. (2018). Orthopaedic manifestations within the 22q11.2 deletion syndrome: a systematic review. Am. J. Med. Genet..

[bib11] Kempen J.H. (2011). Appropriate use and reporting of uncontrolled case series in the medical literature. Am. J. Ophthalmol..

[bib12] Konen O., Armstrong D., Clarke H., Padfield N., Weksberg R., Blaser S. (2008). C1-2 vertebral anomalies in 22q11.2 microdeletion syndrome. Pediatr. Radiol..

[bib13] McDonald-McGinn D.M., Sullivan K.E., Marino B., Philip N., Swillen A., Vorstman J.A.S. (2015). 22q11.2 deletion syndrome. Nat. Rev. Dis. Primers.

[bib14] Morishita Y., Falakassa J., Naito M., Hymanson H.J., Taghavi C., Wang J.C. (2009). The kinematic relationships of the upper cervical spine. Spine (Phila Pa 1976).

[bib15] Óskarsdóttir S., Boot E., Crowley T.B., Loo J.C.Y., Arganbright J.M., Armando M. (2023). Updated clinical practice recommendations for managing children with 22q11.2 deletion syndrome. Genet. Med..

[bib16] Ricchetti E.T., Hosalkar H.S., Gholve P.A., Cameron D.B., Drummond D.S. (2008). Advanced imaging of the cervical spine and spinal cord in 22q11.2 deletion syndrome: Age-matched, double-cohort, controlled study. J Child Orthop.

[bib17] Warner W.C., Azar F.M., Beaty J.H. (2021). Campbell's Operative Orthopaedics [Internet].

